# Omega-3 attenuates the severity of medication-related osteonecrosis of the jaws in rats treated with zoledronate

**DOI:** 10.1371/journal.pone.0320413

**Published:** 2025-03-26

**Authors:** Juliano Milanezi de Almeida, Halef Diego Turini, Henrique Rinaldi Matheus, Otávio Augusto Pacheco Vitória, Bianca Rafaeli Piovezan, Ruan Henrique Barra Dalmonica, Elisa Mara de Abreu Furquim, Edilson Ervolino

**Affiliations:** 1 Periodontics Division, Department of Diagnosis and Surgery, School of Dentistry, São Paulo State University (Unesp), Araçatuba, São Paulo, Brazil; 2 Nucleus of Study and Research in Periodontics and Implantology (NEPPI), School of Dentistry, São Paulo State University (Unesp), Araçatuba, São Paulo, Brazil; 3 Division of Periodontology, College of Dentistry, University of São Paulo, São Paulo, São Paulo, Brazil; 4 Department of Basic Science, School of Dentistry, São Paulo State University (Unesp), Araçatuba, São Paulo, Brazil; Nanjing Medical University, CHINA

## Abstract

This study aimed to evaluate the ability of ω-3 to modulate the tissue response in rats with MRONJ, focusing on histopathological and immunohistochemical parameters. Forty Wistar rats were subjected to bilateral ovariectomy and, three months later, the medication regimen with ZOL (100μg/kg; groups ZOL and ZOL-ω3) of vehicle (VEH and VEH-ω3) was initiated. Following 3 weeks of ZOL or VEH, experimental periodontitis was induced around the mandibular left first molars of all animals. Then, 14 days later (one day before tooth extraction), daily dietary supplementation with ω-3 was given to animals belonging to groups VEH-ω3 or ZOL-ω3. Euthanasia was performed 21 days after tooth extraction. Histologic, histometric (newly-formed bone tissue [NFBT] and non-vital bone tissue [NVBT]), and immunohistochemical (TNF-α, α-SMA, ALP, IL-1β, VEGF, OCN, and TRAP) analyses were performed. Dietary supplementation with ω-3 reduced the amount of NVBT and controlled the intensity and extension of the inflammatory infiltrate in ZOL-ω3, as compared with ZOL. Osteoclast and osteoblast activity were not statistically different between groups ZOL and ZOL-ω3. The structure of the epithelium and the underlining connective tissue were improved by the supplementation with ω-3 in animals under ZOL therapy. Oral supplementation with omega-3 controlled the inflammation and reduced the amount of non-vital bone at the tooth extraction site of ovariectomized rats treated with ZOL and attenuating the severity of MRONJ.

## Introduction

Unlike the 9:1 ratio of medication-related osteonecrosis of the jaw (MRONJ) between cancer and osteoporotic patients estimated in the past [1], a monocentric retrospective study conducted from December 2004 to March 2021 reported that 40% of cases occur in osteoporotic patients [2]. The recent data by Debiève et al. raises awareness of a dramatic concern that antiresorptive drugs may favor MRONJ regardless of prescription (cancer or osteoporosis) [2]. Although multiple drugs are related to this adverse event, an assessment of the Food and Drug Administration Adverse Event Reporting System Database found the 20 most common medications leading to MRONJ reports, of which Zoledronate (ZOL) represented 35.1% of cases [3].

Since American Association of Oral and Maxillofacial Surgeons (AAOMS) position paper in 2014 [4], clinical and animal data have significantly widened the knowledge of the pathophysiology of MRONJ, however, remaining debatable [5]. Bone remodeling inhibition, inflammation or infection, angiogenesis inhibition, innate or acquired immune dysfunction, and genetic predisposition are included in the leading hypothesis unraveling the disease specificity unique to the jaws [4–7].

Modulating hosts inflammatory and/or immune systems effectively address conditions driven or perpetuated by inflammation [8,9]. A new category of modulators, the resolvins, do not suppress acute inflammation (essential to promote optimal healing) [10], but they do prevent its prolongation [11,12]. These immunoresolvents include derivatives omega (ω)-3 fatty acids, the eicosapentaenoic (EPA) and docosahexaenoic (DHA) acids [13,14]. Mediators of inflammation resolution exhibit specialized function on multiple types of cells rather than merely controlling white blood cell function. They exhibit control of stem cells that differentiate into fibroblasts and osteoblasts [15,16], as well as receptor‐mediated control of osteoclasts [17,18]. Additionally, specialized pro-resolvin lipid mediators regulate macrophages towards a pro-healing phenotype (M2-like) over pro-inflammatory (M1-like) activation [19].

The increased risk of MRONJ in patients with periodontal diseases underlines the role of periodontal pathogens in the development of this condition [20]. Interestingly, EPA and DHA significantly reduced the strains of pathogens in an in vitro multi-species subgingival biofilm model [21]. Also, ω-3 may positively act on the inhibited vascularization component of the pathogenesis of MRONJ since a fish oil-enriched diet is associated with improved neovascularization in response to ischemia [22].

Early diagnosis and prevention of MRONJ are paramount to mitigate the need for more invasive surgical interventions required at advanced stages [23–26]. Owing to this rationale, a significant number of preventive approaches, such as ozone therapy [27], mesenchymal stromal cell sheets [28], Sildenafil [29], strontium ranelate [30], and pentoxifylline and α-tocopherol [31], have been proposed to prevent MRONJ. Therefore, considering that ω-3 can act positively on various agents in the pathophysiology of MRONJ, this study aimed to evaluate the ability of ω-3 to modulate the tissue response in rats with MRONJ, focusing on histopathological and immunohistochemical parameters.

## Materials and methods

### Animals

Forty four-month-old female rats (*Rattus norvegicus, albinus*, Wistar) weighing 300-350g were kept under 12/12h light/dark cycles, 22°C ± 2°C ambient temperature, 20 air changes per hour and air humidity about 55% ± 5% and housed in plastic cages, receiving feed and water ad libitum. The experimental protocol was approved by the Ethics Committee in Animal Use (#299-2021) at the School of Dentistry, São Paulo State University (UNESP), Araçatuba. This study was conducted according to the ARRIVE Guidelines [32].

### Sample size calculation

The sample size was estimated according to the previous literature experience [28,29]. The estimation for each outcome measure was performed to achieve a 0.8 power and 0.05 alpha error. Ervolino et al., [33] showed that 7 animals were enough to assure statistical significance for histology, histometry, and immunohistochemistry. To compensate for possible dropouts, 10 animals per group used.

### Randomization method

The study following a single-blind, randomized, controlled design. Numbers from 1 to 40 were labeled in the upper tail of animals. A blinded staff external to the study uploaded the number sequence to the software Minitab® 17 (Minitab Inc., State College, PA, USA) to generate a randomization table for allocating (1:1) the animals to groups vehicle (VEH), VEH-ω3, ZOL, and ZOL-ω3.

### Experimental design

Bilateral ovariectomy (OVX) was performed in all animals 3 months prior to initiating the drug regimen with ZOL (Sigma-Aldrich, MO, EUA) or VEH (Equiplex Indústria Farmacêutica, Aparecida de Goiânia, GO, Brazil). Following OVX and estrous cycle completed, the treatment with vehicle (VEH and VEH-ω3) or ZOL (ZOL and ZOL-ω3) was performed every three days during 3 weeks. The vehicle consisted of 0.45ml (which corresponds to the volume of ZOL administered) [34] of 0.9% sodium chloride solution. The ZOL dose was 100 μg/kg, diluted in 0,45mL of the vehicle, based on human protocols adapted to rats [34,35].

**Fig 1:**
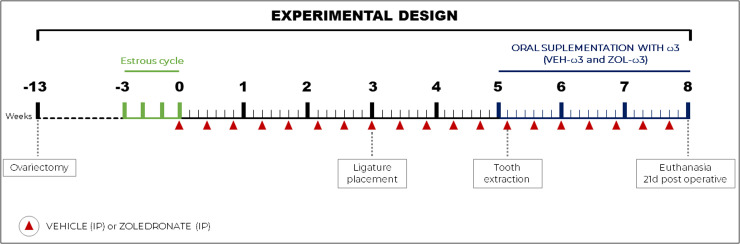
Experimental design. Experiment design timeline showing periods of application of substances, periods of euthanasia and group divisions.

Three weeks after initiating the drug regimen, experimental periodontitis (EP) was induced in all animals by a blinded staff (O.A.P.V). To induce PE, after general anesthesia, the animals were positioned on the operating table to allow adequate access to the oral cavity as well as to favor the maintenance of mouth opening, after which a #24 cotton thread (Coats Corrente, São Paulo, SP, Brazil) was placed subgingivally around the first mandibular molar on the left side using an adapted instrument and held in position by simple surgical knots in the mesial region (free face), favoring the accumulation of bacterial plaque [36,37]. Fourteen days after EP, was initiated the daily supplementation with ω3 (VEH-ω3 and ZOL-ω3) or distilled water (VEH and ZOL) via gastric gavage (GG). The next day, tooth extraction was performed in all animals (Fig 1).

### Sedation and general anesthesia

The surgical interventions were stated by sedation and general anesthesia, obtained by the combination of 70 mg/kg of ketamine hydrochloride (Francota®, Virbac, SP, Brazil) and 6 mg/kg of xylazine hydrochloride (Rompum®, Bayer, RS, Brazil), intramuscularly.

### Ovariectomy and estrous cycle

After a septic preparation, bilateral ovariectomy was performed in all animals (O.A.P.V). For that, bilateral 10 mm incisions were performed caudal to the last pair of ribs aiming to gain access to the abdominal cavity. Then, the ovaries were removed along with a safety margin. Wound closure was obtained by suture on two planes, with resorbable (5-0 Vicryl®, Ethicon Inc, Johnson & Johnson, USA) and non-resorbable threads (5-0 Nylon, Ethicon Inc, Johnson & Johnson, USA) [38]. Each animal received post-surgical intramuscular injections of 24 000 IU of penicillin G-benzathine (Zoetis, SP, Brazil). The daily cytological examination was performed in all animals to determine their cycle phase in the 3 months preceding the beginning of the drug treatment plan. The cytological examination was performed based on the study of Marcondes et al. [39] by quantifying epithelial cells, cornified cells, and leukocytes.

### Protocol for supplementation with omega-3

ω-3 and its solvent were administered daily, via GG. Animals belonging to groups VEH-ω3 and ZOL-ω3 received 40 mg/kg of ω3 (ω3 of animal origin - salmon; 60% EPA and 40% DHA; DrogaVET Bauru - Farmácia de Manipulação Veterinária SA, Bauru, SP, Brazil) [40]. The solvent (distilled water; 0.2 mL) was given to animals from groups VEH and ZOL.

### Tooth extraction

Tooth extraction was carefully performed in all animals by a blinded staff (R.H.B.D). For that, the animals were positioned and 10% povidone‐iodine (Rioquimica, São José do Rio Preto, SP, Brazil) was used for antisepsis of the oral cavity. Syndesmotomy, luxation, and extraction of the mandibular left first molar were performed by means of adapted surgical tools [41]. Sutures with resorbable threads were performed to achieve primary closure of all extraction sockets (5-0 Vicryl®, Ethicon Inc, Johnson & Johnson, USA).

### Euthanasia and sample processing

The animals were euthanized 21 days after the tooth extraction, which was carried out under strict technical and ethical control. The method used for euthanasia was chemical, by administering an excessive dose of anesthetic, following the guidelines established by regulatory bodies such as the National Council for the Control of Animal Experimentation (CONCEA/Brazil) and the American Veterinary Medical Association (AVMA) [42]. The euthanasia procedure was carried out with a lethal dose of Thiopental Sodium (150 mg/kg; Cristália Ltda., Itapira, SP, Brazil), after general anesthesia. Considering that the Thiopental solution has an alkaline pH and can therefore cause irritation or pain, lidocaine (4mg/kg; Bravet, Rio de Janeiro, RJ, Brazil) was added to the solution. The substances were injected intraperitoneally after fasting [42]. To confirm the animal’s death, complementary methods were used to assess vital signs, such as checking for the absence of cardiac, respiratory and pupillary reflexes and any residual brain activity [42].

The left mandibles were collected and fixed in 4% buffered formaldehyde solution for 48 hours. After fixation, all samples were demineralized in 10% EDTA (EDTA; Ethylenediaminetretraacetic acid, Sigma-Aldrich Co.) buffered for 60 days. Subsequently, paraffin embedding was carried out. Sections 4μm thick from the extraction socket (previously occupied by the roots) were obtained in the sagittal plane using microtomy, which the sections were collected on conventional glass microscope slides (Olen®, Kasvi Produtos Laboratory Products), impregnated with polylysine, and on silanized glass slides for microscopy (Platinum Line® StarFrost, Mercedes Medical LLC., Sarasota/FL, USA). Six equidistant sections comprising the furcation from each specimen were stained with hematoxylin and eosin (HE) for histological and histometric analyses of newly formed bone area (BA), and ten other equidistant sections (two for each biomarker), also comprising the central portion of the furcation, were submitted to immunohistochemistry for the detection of tartrate-resistant acid phosphatase (TRAP), tumor necrosis factor (TNF)-α, interleukin (IL)-1β, vascular endothelial growth factor (VEGF), alkaline phosphatase (ALP), α-smooth muscle actin (α-SMA) and osteocalcin (OCN).

The indirect immunoperoxidase technique was performed as described in Matheus et al. [43] Following deparaffinization and hydration, antigen retrieval was performed with the heat-induced epitope retrieval (HIER) method, by immersing the histological slides in citrate buffer solution (Diva decloaker, Biocare Medical, Concord, CA, EUA) in pressurized chamber (Decloaking chamber, Biocare Medical, Concord, CA, EUA) at 95^o^ for 20 minutes. Following, blocking of endogenous peroxidase and non-specific binding sites were done using 3% hydrogen peroxide for 1 h and 1% bovine serum albumin for 12 h, respectively. Next, the sections were divided into four batches, each of them incubated with one of the following primary antibodies: anti-TNFα (1:100; orb11495; Biorbyt, Cambridge, United Kingdom), anti-IL1β (1:100; orb382131; Biorbyt), anti-VEGF (1:100; orb191500; Biorbyt, Cambridge, United Kingdom), anti-OCN (1:100; orb259644; Biorbyt, Cambridge, United Kingdom), anti-ALP (1:100; orb1563120; Biorbyt, Cambridge, United Kingdom), anti-αSMA (1:100; orb704355; Biorbyt, Cambridge, United Kingdom) and anti-TRAP (1:200; orb2250; Biorbyt, Cambridge, United Kingdom). For signal amplification, universal biotinylated secondary antibodies (1:200; anti-goat IgG +  anti-rabbit IgG +  anti-mouse IgG) and streptavidin conjugated with horseradish peroxidase (HRP) (Universal Dako Labeled HRP Streptavidin-Biotin Kit, Dako Laboratories, CA, EUA) were used. The reaction was developed using the chromogen 3,3′-diaminobenzidine tetrahydrochloride (ImmPACT DAB Substrate, Vector Laboratories). Lastly, Harris Hematoxylin was used as counterstaining.

### Analysis of the results

The histopathologic and histometric analyses were performed by staffs masked to the treatments performed (H.D.T. and E.E., respectively). A digital camera (AxioCam MRc5, Carl Zeiss Microscopy GmbH, NI, Germany) coupled to a light microscope (Axio Scope, Carl Zeiss Microscopy GmbH, NI, Germany) was used to record the regions of interest (ROI) [33]. In summary, ROI consisted of a 4mm × 4mm area which included the portion of the tooth extraction site formerly occupied by the mesial and distal roots of the lower left first molar and adjacent tissues.

### Histopathological analysis of the extraction site and adjacent tissues

The histologic analysis evaluated the following parameters: 0) intensity of local inflammatory response; 1) extension of inflammatory process; 2) cellular and structure pattern of epithelial tissue; 3) cellular and structure pattern of connective tissue; 4) cellular and structure pattern of bone and tissue.

### Histometric analysis of NFBT and NVBT

Image analysis software ImageJ (ImageJ, U. S. National Institutes of Health, Bethesda, Maryland, USA) was used to measure the total amount of bone. Then, the same software was used to determine NFBT and NVBT. The results for both outcome measures are expressed as the percentage of area occupied by vital (NFBT) or non-vital (NVBT). It was considered non-vital bone when ten or more neighboring lacunae of osteocytes were empty or containing necrotic remnants of osteocytes [44].

### Immunohistochemical analysis

The immunohistochemical analysis for all markers was performed by a certified histologist masked to the experimental groups (E.E). Were considered immunoreactive (IR) cells the ones presenting dark brown coloration confined to the cytosolic compartment (TRAP) or confined to the cytosolic compartment and poorly to the extracellular matrix (TNF-α, ALP, α-SMA, IL-1β, VEGF, and OCN).

A semi-quantitative analysis of the immunolabeling of TNF-α, ALP, α-SMA, IL-1β, VEGF, and OCN was performed based on the criteria of Gusman et al., [45] as follows:

- Score 0: no immunolabeling (total absence of IR cells);
- Score 1: low immunolabeling (IR in ∼ 1/4 of cells per area);
- Score 2: moderate immunolabeling (IR in ∼ 1/2 of cells per area);
- Score 3: high immunolabeling (IR in ∼ 3/4 of cells per area).

For TRAP analysis, the following parameters were considered:

- Score 0: total absence of IR cells;
- Score 1: 1 – 6 immunolabeling cells/mm²;
- Score 2: 7 – 12 immunolabeling cells/mm²;
- Score 3: more than 12 immunolabeling cell/mm².

### Primary and secondary outcomes

The primary outcome was defined as the amount of non-vital bone (NVBT) at the post-extraction socket. Secondary results are related to histopathological features and immunohistochemical results.

### Statistical analysis

Data were analyzed using BioStat (BioStat version 5.0, Belém, PA, Brazil). Shapiro-Wilk test was used to assess the normality of the data. For TRAP, TNF-α, ALP, α-SMA, IL-1β, VEGF, and OCN, the significance of the differences among groups was determined by a Kruskal‒Wallis test, followed by a post-hoc Student‒Newman‒Keuls test (p ≤ 0.05). For NFBT and NVBT, the significance of differences among groups was determined by one-way analysis of variance (ANOVA), followed by a post-hoc Tukey test (p ≤ 0.05).

## Results

All ligatures were maintained for 14 days (no ligatures were lost). In four animals, the apical third of the root fractured and the fragment was removed using non-traumatic instruments. All the data is available in the support information section (S1 File).

### Intra-oral and tooth extraction site clinical features

No macroscopic differences were observed in the oral clinical examination at the extraction site for the VEH, VEH-ω3 and ZOL-ω3 groups. However, some animals in the ZOL group showed areas of bone exposure at the extraction site, consistent with the pathological characteristics of MRONJ and confirmed by the histopathological results, our primary variable.

### Percentage of NFBT and NVBT in tooth extraction sites

**Fig 2:**
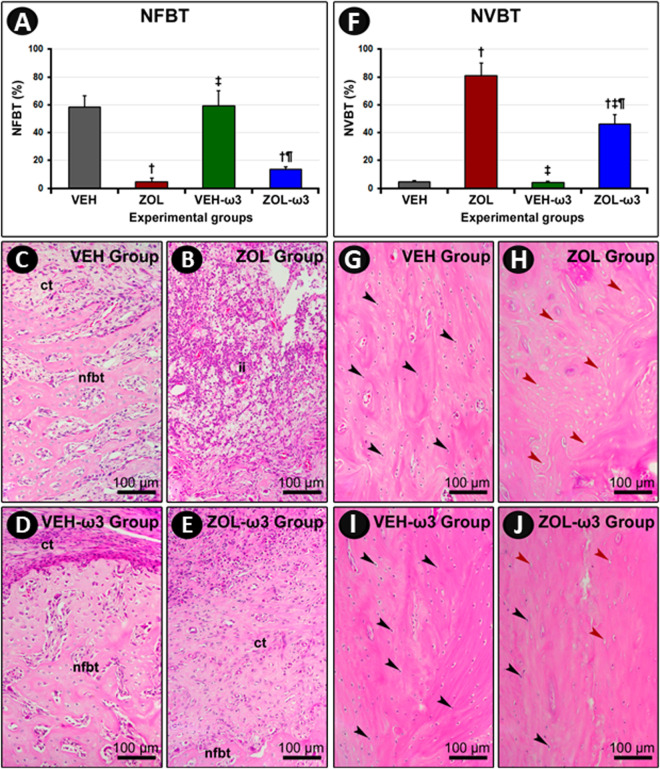
Vital (NVBT) and non-vital bone tissue in the tooth (NFBT) extraction site. (A) Percentage of newly formed bone tissue (NFBT), (ct): connective tissue and (ii) inflammatory infiltrate in the extraction site in the different experimental groups at 21 postoperative days. (F) percentage of non-vital bone tissue (NVBT) in extraction site in the different experimental groups at 21 postoperative days. Black arrows indicate regions where the lacunae are occupied by viable osteocytes. Red arrows indicate regions where the lacunae are completely empty of occupied the remains of osteocytes. Statistical tests: Tukey test followed by post-hoc Tukey test (P ≤ 0.05). (B–E) Photomicrographs showing percentage of NFBT in extraction site previously occupied by mesial root of first molar in groups VEH (c), ZOL (b), VEH-ω3 (d) and ZOL-ω3 (e). (G–J) Photomicrographs showing percentage of NVBT in extraction site previously occupied by mesial root of first molar in groups VEH (g), ZOL (h), VEH-ω3 (i) and ZOL-ω3 (j). Staining: HE. Scale bars: 100 μm. Source: from the authors themselves. (†) statistically significant difference with group VEH; (‡) statistically significant difference with group ZOL; (¶) statistically significant difference with group VEH-ω3.

The groups treated with ZOL (ZOL and ZOL-ω3) exhibited lower percentage of NFBT when compared with groups treated with VEH (VEH and VEH-ω3). NFBT did not differ between groups VEH and VEH-ω3, neither between ZOL and ZOL-ω3 (Fig 2). The ZOL group exhibited the highest percentage of NVBT as compared with all other groups (ZOL-ω3, VEH, and VEH-ω3). The percentage of NVBT was lower in ZOL-ω3 as compared with ZOL. The percentage of NVBT was higher than in groups treated with VEH (VEH and VEH-ω3). No statistically significant difference in the percentage of NVBT when comparing VEH and VEH-ω3 (Fig 2).

### Histopathological analysis of the tooth extraction site and adjacent tissues

When considering the intensity and extension of the inflammatory response, the specimens from group ZOL-ω3 were almost evenly distributed within scores 1 and 2, while in group ZOL most specimens presented a large amount of inflammatory cells (score 3). Also, while in group ZOL-ω3 most specimens presented an inflammatory process confined to the connective tissue (score 2), in ZOL the inflammatory process extended to the whole connective tissue and to the alveolar bone (Tables 1 and 2).

**Table 1:** Scores and specimens’ distribution according to the parameters of the histopathologic analysis assessing the levels of inflammation at the tooth extraction site and adjacent tissues.

Histopathologic analysis: Level of inflammation				
Scores for each parameter	Percentage of specimens			
	Experimental groups			
	VEH	ZOL	VEH-ω3	ZOL-ω3
**Intensity of local inflammatory response**				
**(0)** absence of inflammation	100%	–	100%	–
**(1)** small amount of inflammatory cells (less than 1/3 of the cells are inflammatory)	–	–	–	60%
**(2)** moderate amount of inflammatory cells (between 1/3 and 2/3 of the cells are inflammatory)	–	20%	–	40%
**(3)** large amount of inflammatory cells (more than 2/3 of the cells are inflammatory)	–	80%	–	–
**Median**	**1**	**4** ^†^	**1** ^‡^	**2** ^†^ ^,^ ^¶^
**Extension of the inflammatory process**				
**(0)** absence of inflammation	100%	–	100%	–
**(1)** extending to part of the connective tissue	–	–	–	20%
**(2)** extending to the whole connective tissue	–	20%	–	80%
**(3)** extending to the whole connective tissue and to the alveolar bone	–	80%	–	–
**Median**	**1**	**4** ^†^	**1** ^‡^	**2** ^†^ ^,^ ^¶^

Statistical tests: Kruskal–Wallis test followed by post-hoc Student Newman–Keuls test (P ≤ 0.05). Symbols:

† , statistically significant difference with group VEH;

‡ , statistically significant difference with group ZOL;

¶ , statistically significant difference with group VEH-ω3.

**Table 2:** Scores and specimens’ distribution according to the parameters of the histopathologic analysis assessing the structural organization of the tooth extraction site and adjacent tissues.

Histopathologic analysis: Structural pattern				
Scores for each parameter	Percentage of specimens			
	Experimental groups			
	VEH	ZOL	VEH-ω3	ZOL-ω3
**Structural pattern of the underlining epithelial tissue**				
**(0)** epithelial tissue with moderate thickness completely overlining the tooth extraction site	40%	–	60%	–
**(1)** thin epithelial tissue completely overlining the tooth extraction site	60%	–	40%	40%
**(2)** thin epithelial tissue overlining strictly the edged of the tooth extraction site	–	–	–	60%
**(3)** absence of epitelial tissue overlining the tooth extraction site	–	100%	–	–
**Median**	**2**	**4** ^†^	**1** ^‡^	**3** ^†^ ^,^ ^‡^ ^,^ ^¶^
**Pattern of the connective tissue structure**				
**(0)** moderate amount of fibroblasts and large amount of collagen fibers (dense connective tissue)	60%	–	60%	–
**(1)** moderate amount of fibroblasts and collagen fibers	40%	–	40%	20%
**(2)** small amount of fibroblasts and collagen fibers	–	–	–	80%
**(3)** severe tissue breakdown and areas with necrosis	–	100%	–	–
**Median**	**1**	**4** ^†^	**1** ^‡^	**3** ^†^ ^,^ ^‡^ ^,^ ^¶^
**Pattern of the alveolar bone structure**				
**(0)** absence of non-vital bone in adjacencies of extraction site and trabecular bone filling more than half of tooth socket	100%	–	100%	–
**(1)** absence of non-vital bone in adjacencies of extraction site and trabecular bone filling less than half of tooth socket	–	–	–	20%
**(2)** presence of few areas with non-vital bone in adjacencies of extraction site and trabecular bone filling less than a third of tooth socket	–	–	–	80%
**(3)** presence of many areas with non-vital bone in adjacencies of extraction site and trabecular bone filling less than a third of tooth socket	–	100%	–	–
**Median**	**1**	**4** ^†^	**1** ^‡^	**3** ^†^ ^,^ ^¶^

Statistical tests: Kruskal–Wallis test followed by post-hoc Student Newman–Keuls test (P ≤ 0.05). Symbols:

† , statistically significant difference with group VEH;

‡ , statistically significant difference with group ZOL;

¶ , statistically significant difference with group VEH-ω3.

In ZOL-ω3, the specimens were distributed between scores 1 and 2, with most animals exhibiting thin epithelial tissue overlining strictly the edged of the tooth extraction site. On the other hand, in group ZOL, all specimens presented complete absence of epithelial tissue overlining the tooth extraction. Similar distribution of the specimens from groups ZOL-ω3 and ZOL to each score was observed when evaluating the connective tissue and the alveolar bone structure. For these parameters, all specimens from group ZOL were included in score 3, while most specimens from group ZOL-ω3 were in score 2 (Tables 1 and 2).

### Immunolabeling pattern for TNF-α and IL-1β in tooth extraction sites

**Fig 3:**
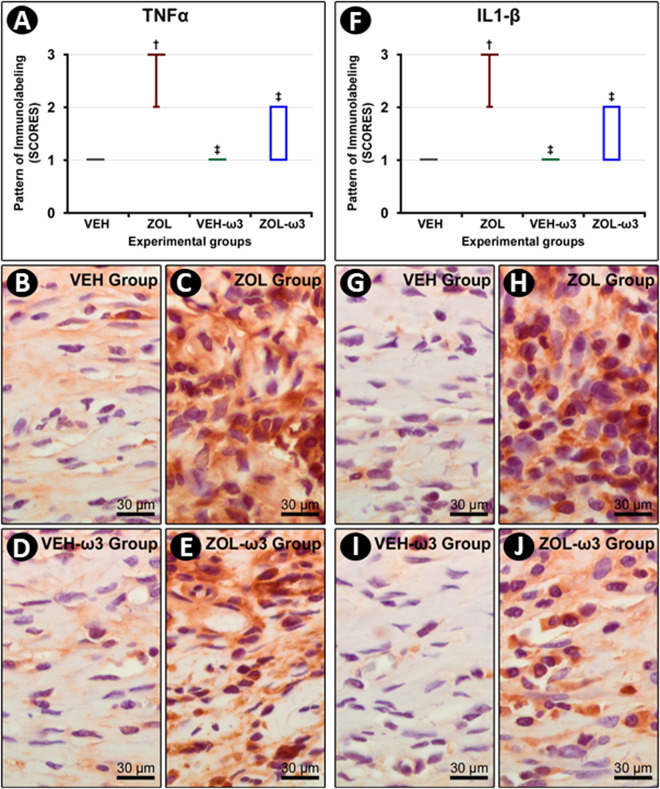
Immunolabeling pattern for TNF-α and IL-1β in the tooth extraction site. (A) Graph showing the median and interquartile range of the scores for TNF-α in the furcation for each group and period. (F) Graph showing the median and interquartile range of the scores for IL-1β in the furcation for each group and period. Statistical tests: Kruskal–Wallis test followed by post-hoc Student Newman–Keuls test (P ≤ 0.05). (B–E) Photomicrographs showing immunolabeling pattern for TNF-α in the tooth extraction site in groups VEH (B), ZOL (C), VEH-ω3 (D) and ZOL-ω3 (E). (G–J) Photomicrographs showing immunolabeling pattern for IL-1β in the tooth extraction site in groups VEH (G), ZOL (H), VEH-ω3 (I) and ZOL-ω3 (J). Counterstaining: Harry’s Hematoxylin. Scale bars: 30 μm. Source: from the authors themselves. (†) statistically significant difference with group VEH; (‡), statistically significant difference with group ZOL.

The immunolabeling for TNF-α and IL-1β was predominantly observed in inflammatory cells and extracellular matrix (Fig 3). The ZOL group exhibited higher immunolabeling pattern for both TNF-α and IL-1β as compared with all other groups (ZOL-ω3, VEH, and VEH-ω3). The immunolabeling patter for TNF-α and IL-1β was lower in ZOL-ω3 as compared with ZOL. No statistically significant differences were observed regarding the immunolabeling for TNF-α and IL-1β between VEH and VEH-ω3 (Fig 3).

### Immunolabeling pattern for ALP and OCN in tooth extraction sites

**Fig 4:**
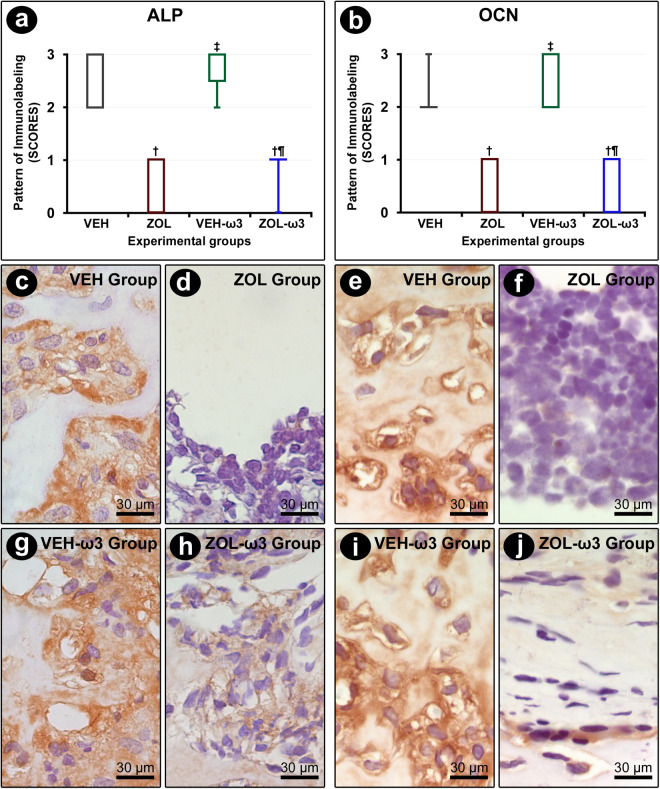
Immunolabeling pattern for ALP and OCN in the tooth extraction site. (A, B) Graph showing the median and interquartile range of the scores for ALP (A) and OCN (B) in the furcation for each group and period. Statistical tests: Kruskal–Wallis test followed by post-hoc Student Newman–Keuls test (P ≤ 0.05). (A, B) Photomicrographs showing immunolabeling pattern for ALP (C, D, G, H) and OCN (E, F, I, J) in the tooth extraction site in groups VEH (C, E), ZOL (D, F), VEH-ω3 (G, I) and ZOL-ω3 (H, J). Counterstaining: Harry’s Hematoxylin. Scale bars: 30 μm. Source: from the authors themselves. (†) statistically significant difference with group VEH; (‡) statistically significant difference with group ZOL; (¶) statistically significant difference with group VEH-ω3.

ALP and OCN immunolabeling was observed in osteoblasts. The immunolabeling pattern for ALP and OCN was lower in ZOL and ZOL-ω3 as compared with their controls, VEH and VEH-ω3, respectively. No statistically significant differences were observed regarding the immunolabeling for ALP and OCN when comparing VEH and VEH-ω3 or ZOL and ZOL-ω3 (Fig 4).

### Immunolabeling pattern for VEGF and αSMA in tooth extraction sites

**Fig 5:**
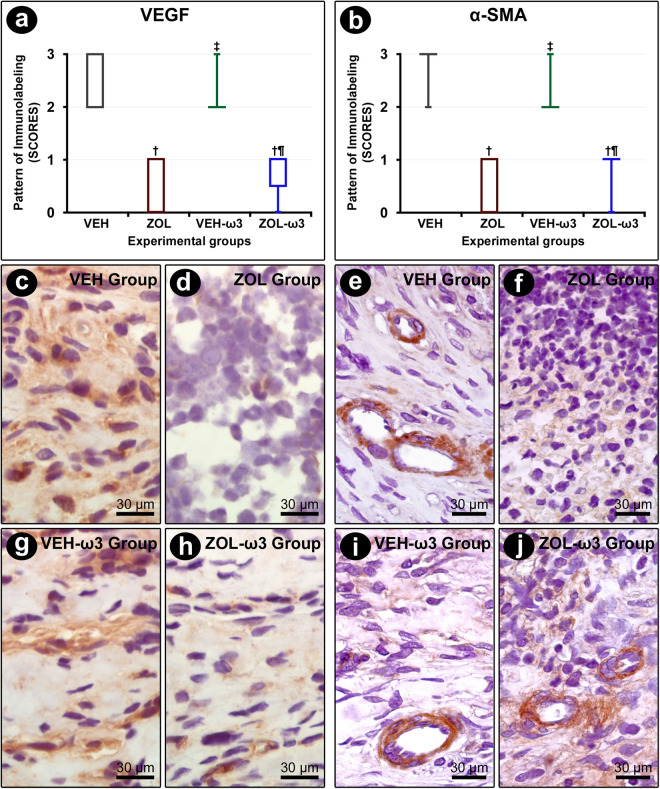
Immunolabeling pattern for VEGF and αSMA in the tooth extraction site. (A, B) Graph showing the median and interquartile range of the scores for VEGF (A) and αSMA (B) in the furcation for each group and period. Statistical tests: Kruskal–Wallis test followed by post-hoc Student Newman–Keuls test (P ≤ 0.05). (A, B): Photomicrographs showing immunolabeling pattern for VEGF (C,D,G,H) and αSMA (E, F, I, J) in the tooth extraction site in groups VEH (C, E), ZOL (D, F), VEH-ω3 (G,I) and ZOL-ω3 (H, J). Counterstaining: Harry’s Hematoxylin. Scale bars: 30 μm. Source: from the authors themselves. (†) statistically significant difference with group VEH; (‡) statistically significant difference with group ZOL; (¶) statistically significant difference with group VEH-ω3.

VEGF immunolabeling was mainly observed in fibroblasts and osteoblasts, while α-SMA immunolabeling was confined to

myofibroblast. The immunolabeling pattern for VEGF and αSMA was lower in ZOL and ZOL-ω3 as compared with their controls, VEH and VEH-ω3, respectively. No statistically significant differences were observed regarding the immunolabeling for VEGF and αSMA when comparing VEH and VEH-ω3 or ZOL and ZOL-ω3 (Fig 5).

### TRAP in tooth extraction sites

**Fig 6:**
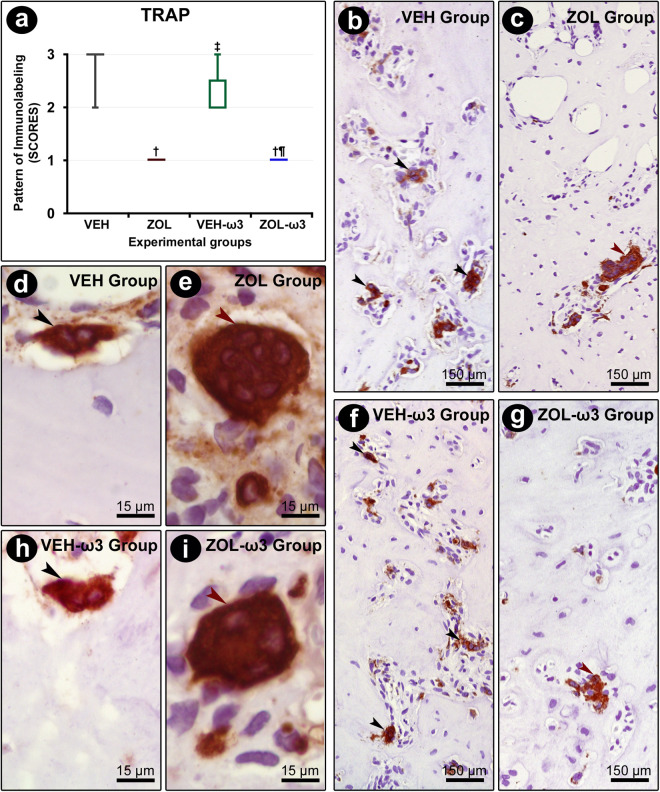
Immunolabeling pattern for TRAP in the tooth extraction site. (A) Graph showing the median and interquartile range of the scores for TRAP in the furcation for each group and period. Black arrows indicate osteoclasts of normal dimensions, which are active, as observed by their coupling to Howship lacunae. Red arrows indicate abnormally huge round-shaped osteoclasts, hypernucleated, and distant from the bone matrix, all features that indicate inactivity. Statistical tests: Kruskal–Wallis test followed by post-hoc Student Newman–Keuls test (P ≤ 0.05). (B–I) Photomicrographs showing immunolabeling pattern for TRAP in the tooth extraction site in groups VEH (B, D), ZOL (C, E), VEH-ω3 (F, H) and ZOL-ω3 (G, I). Counterstaining: Harry’s Hematoxylin. Scale bars: 15 μm and 150 μm. Source: from the authors themselves. (†) statistically significant difference with group VEH; (‡) statistically significant difference with group ZOL; (¶) statistically significant difference with group VEH-ω3.

TRAP immunolabeling was confined to the cytosolic compartment of osteoclasts. The osteoclasts observed in groups treated with ZOL (ZOL and ZOL-ω3) were bigger in size, round, hypernucleated, and considerably distant from the bone matrix (not coupled) (Fig 6). The immunolabeling pattern for TRAP was lower in ZOL and ZOL-ω3 as compared with their controls, VEH and VEH-ω3, respectively. No statistically significant differences were observed regarding the immunolabeling for TRAP when comparing VEH and VEH-ω3 or ZOL and ZOL-ω3 (Fig 6).

## Discussion

Genome-wide and whole-exome sequencing studies have uncovered the genetic component of the pathophysiology of MRONJ [46,47], thus adding this condition to a vast and concerning list of “complex diseases”. Complex diseases are the result of a combination of genetic, environmental, and lifestyle factors [48], most of which have not yet been identified. Achieving optimal prevention or therapeutic success when dealing with simultaneous and overlapping inflammatory/immune, vascular, microbial, environmental (medications), and genetic factors is a great challenge to researchers and clinicians. In this study, although not preventing the occurrence of MRONJ in all animals, ω-3 supplementation significantly reduced the amount of NVBT in the extraction sites.

Suppression of intracortical remodeling induced by bisphosphonates (BPs) has been widely accepted as a critical player in the pathogenesis of MRONJ [49,50]. Unlike humans and large animals [51], rodents do not feature spontaneous intracortical remodeling, which could limit the value of rats/mice studies investigating this condition. However, OVX alone was capable of inducing intracortical remodeling in skeletally mature mice and rats (3 months old) [52,53], fostering reliable clinical inferences. It is also essential to emphasize equivalency in a plurality of the biological features in the morphology and physiology of pristine alveolar [54] and on tooth extraction socket healing when comparing swine and rodents [55]. In summary, morphofunctional modifications in trabecular bone, along with alterations in indicators related to bone metabolism, were observed in rodents subjected to OVX [38]. These changes include a reduction in levels of OCN, ALP, and procollagen type I N-terminal propeptide (P1NP), an increase in C-terminal telopeptide of type I collagen (CTX1), a decrease in bone mineral density, and a reduction in mandibular and long bone volume [38,53,56]. These factors establish significant alterations in bone turnover. Furthermore, there was a reduction in vascularization and changes in biomechanical parameters. Song et al., [38] demonstrated that the period between 8 and 16 weeks post-OVX in rats is consistent with the development of osteoporotic characteristics. These characteristics arise from estrogen deficiency and include intracortical remodeling. In the present experiment, analyses were initiated 13 weeks after OVX. Hence, aiming to closely emulate an ideal clinical scenario for developing MRONJ, our rat preclinical model combined activation of intracortical bone remodeling in rats (achieved with OVX) with high dosages of ZOL and local risk factors (periodontitis and tooth extraction) [57].

As previously mentioned, periodontitis and tooth extraction are considered local risk factors that directly influence the pathophysiology of MRONJ [58]. In ligature-induced periodontitis, as employed in this study, the positioning and type of ligature used lead to bacterial plaque adherence and ulceration of the sulcular epithelium, facilitating bacterial penetration into epithelial and connective tissues, alongside the apical migration of the junctional epithelium [59–61]. This process promotes uncontrolled immunoinflammatory response, resulting in the loss of periodontal ligament fibers and, consequently, the destruction of bone tissue [59–61]. Considering the synergistic characteristics of the pathophysiology of periodontitis associated with tooth extraction and the exacerbation of MRONJ, previous studies, such as that of Aghaloo et al., [62] observed an increase in MRONJ associated with ligature-induced periodontitis in rats, particularly in groups treated with high doses of ZOL, demonstrating significant bone tissue disruption through the intensification of the inflammatory response and the presence of exposed necrotic bone. Furthermore, the deterioration of periodontal condition often necessitates dental extractions, a procedure linked to a higher incidence of MRONJ. Soundia et al., [63] demonstrated that in rats with induced periodontitis combined with subsequent tooth extraction and exposure to ZOL, a persistent inflammatory infiltrate, a greater amount of necrotic bone tissue, and intensified collagenolytic activity were observed, findings supported by Zhang et al., [64] who highlighted that when tooth extraction is performed in the presence of periodontitis, there is an increased incidence of MRONJ.

The mechanism of MRONJ has not been fully understood so far, however, the participation of intense and persistent inflammatory infiltration in MRONJ lesions is irrefutable [5,57,65]. Microarray analysis based on the datasets of high-throughput sequencing is one of the most important technologies for the investigation of the diagnosis methods and pathogenic mechanisms of disease [66–68]. Ma et al. [69] excavated the biological information of the chips in GEO database to identify critical factors of osteonecrosis via analyzing the gene network and revealing signaling pathways by enrichment analysis. Their Protein-Protein Interaction Network Analysis showed that the progression of osteonecrosis of the jaw is related to (but not only) TNF and IL-1β. TNF-α and IL-1β are important regulators of the inflammatory response, overexpressed in inflammatory osteolysis [70,71]. The beneficial effects of ω-3 in reducing the severity of MRONJ, reported in this study, might rely on the positive modulation of the local inflammatory infiltrate, evidenced by the low to moderate expression of TNF-α and IL-1β in ZOL-ω3, opposed to the high immunoreactivity of both markers in group ZOL.

There is a confounding issue when determining whether microbial shift or immune response is the trigger for developing periodontitis [72], a major local risk factor for MRONJ [5]. Indeed, one cannot be there without the other. However, the “Inflammation-Mediated Polymicrobial-Emergence and Dysbiotic-Exacerbation” model suggests that the inflammation continuum is the driving force for the selective expansion of periodontal pathogens [73], which only plays a role at a late stage of periodontitis. Studies have supported this hypothesis when reporting the capacity of resolvins, as monotherapy (no scaling and root planning performed), in preventing alveolar bone loss in animal models of periodontitis [74,75]. Also, Serhan et al. [76] compiled evidence on pro-resolving mediators bridging the resolution of infectious inflammation to tissue regeneration. These data suggest that regardless of infection being a primary etiological factor or a result of immune imbalance, the capacity of resolvins or their precursors, ω-3, to govern the resolution of inflammation and tissue repair ought to be exploited in the setting of prevention or treatment of MRONJ.

After circulating BPs are absorbed into the bone, the bone resorption process promoted by osteoclast dissociates BPs from the bone surface and allows intracellular uptake into osteoclasts by fluid phase endocytosis [77]. Inside osteoclast, BPs specifically inhibit farnesyl pyrophosphate synthase, a key branch-point enzyme in the mevalonate pathway, that generates isoprenoid lipids utilized in sterol synthesis and for the post-translational modification of small GTP-binding proteins essential for osteoclast function [78–80]. Although this study focusing on changes restricted to the extraction socket, the potent action of ZOL was evidenced by osteoclasts featuring characteristics of inactivity (large rounded, hypernucleated cells, without cell polarization and distant from the bone matrix), and, corroborating with other experiments, dramatic reduction in the number of TRAP-positive cells/mm^2^ [33,80].

Although the direct toxicity and inhibition of osteoblast differentiation had been already shown [81], Hadad et al., [82] added that ZOL negatively impacts the differentiation of human bone marrow stem cells (hBMSCs) towards the osteoblastic lineage in a dose-dependent manner. Their findings corroborate with Fleifel et al., [83] which reported a bifunctional activity of ZOL, either enhancing osteogenic differentiation and in-vitro mineralization of human mesenchymal stem cells (hMSCs) at low concentration or inducing hMSCs death at high concentrations. The reduced amount of newly-formed bone combined with the lower immunolabeling pattern for OCN and ALP in group ZOL and ZOL-ω3 reinforces that the imbalance in bone turnover in individuals under nitrogen-containing BPs therapy relies on mechanistic changes not restricted to osteoclasts’ activity, which were not restored by supplementing animals with ω-3.

Keratinocytes, fibroblasts, and endothelial cells are essential cell lines that orchestrate alveolar bone repair and are alarmingly compromised by ZOL [63,84,85]. In our study, there was no statistical significance between the immunolabeling pattern of VEGF and αSMA in groups ZOL and ZOL-ω3. However, while animals in group ZOL were equally distributed between scores 0 and 1, most animals in group ZOL-ω3 presented low immunolabeling (score 1). Also, the beneficial properties of ω-3 or its derivatives in keratinocytes [86] and gingival fibroblasts [87] might have supported the improved characteristics of the epithelium, lamina propria, and structuring of collagen fibers in the mucosa overlying the tooth extraction site in ZOL-ω3.

Recently, Hadad et al. [76] accurately stratified the percentage of rat ZOL oncologic dose that corresponds to human use into three categories (physiologic [≤200%], supraphysiologic [>200% <  1000%], and extremely supraphysiologic [>1000%]). Based on their calculation, the dosage used in the present experiment is extremely supraphysiologic. In an animal experiment aimed to create a reliable scenario of the development of MRONJ considering the rapid rat metabolism, it is reasonable to use higher dosages of the drug. However, it might be considered that benefits achievable in a clinical setting are potentially masked by extremely supraphysiologic dosages of ZOL. Given the impact of MRONJ on the quality of life of patients, the promising findings of the use of dietary supplementation with ω-3 on the development of MRONJ, and the emergence of resolution [88,89], we encourage future research assessing specialized pro-resolving mediators for prevention and treatment of MRONJ.

## Conclusion

Oral supplementation with omega-3 controlled the inflammation and reduced the amount of non-vital bone at the tooth extraction site of ovariectomized rats treated with ZOL and attenuating the severity of MRONJ.

## Supporting information

S1 File Availability of data from all analyses. (DOCX)
