# Supplementary material for: Omega-3 attenuates the severity of medication-related osteonecrosis of the jaws in rats treated with zoledronate
Source: PLoS One. 2025 Mar 26;20(3):e0320413. doi: 10.1371/journal.pone.0320413 (PMC11940605; doi:10.1371/journal.pone.0320413)
Supplement: S1 File — (DOCX) [file pone.0320413.s001.docx]

| **VARIABLE**  **1** | **NFBT** | | | |
| --- | --- | --- | --- | --- |
| **GROUPS** | **VEH** | **ZOL** | **VEH-ω3** | **ZOL-ω3** |
| **PERCENTAGES** | **58.67** | **6.12** | **68.99** | **11.14** |
|  | **61.47** | **8.44** | **74.12** | **13.89** |
|  | **49.78** | **2.89** | **62.11** | **16.90** |
|  | **67.98** | **0.97** | **54.78** | **12.80** |
|  | **64.12** | **1.97** | **49.12** | **12.92** |
|  | **60.31** | **6.78** | **44.37** | **13.77** |
|  | **44.97** | **5.01** | **62.11** | **13.90** |
| **MEAN** | **58.2** | **4.6** | **59.4** | **13.6** |
| **STANDARD DEVIATION** | **8.1** | **2.7** | **10.6** | **1.7** |

| **VARIABLE**  **2** | **NVBT** | | | |
| --- | --- | --- | --- | --- |
| **GROUPS** | **VEH** | **ZOL** | **VEH-ω3** | **ZOL-ω3** |
| **PERCENTAGES** | **3.14** | **90.12** | **3.27** | **44.12** |
|  | **5.12** | **74.89** | **2.79** | **39.47** |
|  | **3.21** | **90.13** | **3.99** | **40.97** |
|  | **6.90** | **85.12** | **4.79** | **44.64** |
|  | **4.02** | **80.75** | **5.12** | **50.65** |
|  | **3.98** | **64.22** | **4.08** | **59.70** |
|  | **4.13** | **80.90** | **4.97** | **43.66** |
| **MEAN** | **4.4** | **80.9** | **4.1** | **46.2** |
| **STANDARD DEVIATION** | **1.3** | **9.2** | **0.9** | **6.9** |

| **VARIABLE**  **3** | **TNFα** | | | |
| --- | --- | --- | --- | --- |
| **GROUPS** | **VEH** | **ZOL** | **VEH-ω3** | **ZOL-ω3** |
| **SCORES** | **1** | **3** | **1** | **2** |
|  | **1** | **3** | **1** | **2** |
|  | **1** | **3** | **1** | **2** |
|  | **1** | **2** | **1** | **1** |
|  | **1** | **3** | **1** | **1** |
|  | **1** | **3** | **1** | **2** |
|  | **1** | **3** | **1** | **1** |
| **MEDIAN** | **1** | **3** | **1** | **2** |
| **INTERQUARTILE RANGE** | **(1 - 1)** | **(2 - 3)** | **(1 - 1)** | **(1 - 2)** |

| **VARIABLE**  **4** | **IL-1β** | | | |
| --- | --- | --- | --- | --- |
| **GROUPS** | **VEH** | **ZOL** | **VEH-ω3** | **ZOL-ω3** |
| **SCORES** | **1** | **2** | **1** | **1** |
|  | **1** | **3** | **1** | **1** |
|  | **1** | **3** | **1** | **2** |
|  | **1** | **3** | **1** | **2** |
|  | **1** | **3** | **1** | **1** |
|  | **1** | **3** | **1** | **2** |
|  | **1** | **3** | **1** | **2** |
| **MEDIAN** | **1** | **3** | **1** | **2** |
| **INTERQUARTILE RANGE** | **(1 - 1)** | **(2 - 3)** | **(1 - 1)** | **(1 - 2)** |

| **VARIABLE**  **5** | **VEGF** | | | |
| --- | --- | --- | --- | --- |
| **GROUPS** | **VEH** | **ZOL** | **VEH-ω3** | **ZOL-ω3** |
| **SCORES** | **3** | **1** | **2** | **0** |
|  | **2** | **0** | **2** | **1** |
|  | **2** | **1** | **3** | **1** |
|  | **3** | **0** | **2** | **1** |
|  | **2** | **1** | **2** | **0** |
|  | **2** | **0** | **2** | **1** |
|  | **3** | **0** | **2** | **1** |
| **MEDIAN** | **2** | **0** | **2** | **1** |
| **INTERQUARTILE RANGE** | **(2 - 3)** | **(0 - 1)** | **(2 - 3)** | **(0 - 1)** |

| **VARIABLE**  **6** | **αSMA** | | | |
| --- | --- | --- | --- | --- |
| **GROUPS** | **VEH** | **ZOL** | **VEH-ω3** | **ZOL-ω3** |
| **SCORES** | **3** | **1** | **2** | **1** |
|  | **3** | **0** | **2** | **1** |
|  | **3** | **1** | **3** | **1** |
|  | **3** | **0** | **2** | **1** |
|  | **3** | **1** | **2** | **0** |
|  | **3** | **0** | **2** | **1** |
|  | **2** | **0** | **2** | **1** |
| **MEDIAN** | **3** | **0** | **2** | **1** |
| **INTERQUARTILE RANGE** | **(2 - 3)** | **(0 - 1)** | **(2 - 3)** | **(0 - 1)** |

| **VARIABLE**  **7** | **ALP** | | | |
| --- | --- | --- | --- | --- |
| **GROUPS** | **VEH** | **ZOL** | **VEH-ω3** | **ZOL-ω3** |
| **SCORES** | **2** | **0** | **3** | **0** |
|  | **3** | **0** | **3** | **1** |
|  | **2** | **1** | **3** | **1** |
|  | **2** | **1** | **2** | **1** |
|  | **3** | **1** | **3** | **1** |
|  | **2** | **1** | **2** | **1** |
|  | **3** | **0** | **3** | **1** |
| **MEDIAN** | **2** | **1** | **3** | **1** |
| **INTERQUARTILE RANGE** | **(2 - 3)** | **(0 - 1)** | **(2 - 3)** | **(0 - 1)** |

| **VARIABLE**  **8** | **OCN** | | | |
| --- | --- | --- | --- | --- |
| **GROUPS** | **VEH** | **ZOL** | **VEH-ω3** | **ZOL-ω3** |
| **SCORES** | **2** | **0** | **2** | **0** |
|  | **3** | **0** | **3** | **0** |
|  | **2** | **1** | **3** | **1** |
|  | **2** | **1** | **2** | **0** |
|  | **2** | **1** | **2** | **1** |
|  | **2** | **0** | **2** | **1** |
|  | **2** | **0** | **3** | **1** |
| **MEDIAN** | **2** | **0** | **2** | **1** |
| **INTERQUARTILE RANGE** | **(2 - 3)** | **(0 - 1)** | **(2 - 3)** | **(0 - 1)** |

| **VARIABLE**  **9** | **TRAP** | | | |
| --- | --- | --- | --- | --- |
| **GROUPS** | **VEH** | **ZOL** | **VEH-ω3** | **ZOL-ω3** |
| **SCORES** | **2** | **1** | **2** | **1** |
|  | **2** | **1** | **3** | **1** |
|  | **1** | **1** | **2** | **1** |
|  | **2** | **1** | **3** | **1** |
|  | **2** | **1** | **2** | **1** |
|  | **2** | **1** | **2** | **1** |
|  | **2** | **1** | **2** | **1** |
| **MEDIAN** | **2** | **1** | **2** | **1** |
| **INTERQUARTILE RANGE** | **(1 - 2)** | **(1 - 1)** | **(2 - 3)** | **(1 - 1)** |
